# Bioconversion of Agro-Industrial Byproducts by Applying the Solid-State Fermentation Bioprocess to Increase Their Antioxidant Potency

**DOI:** 10.3390/antiox14080910

**Published:** 2025-07-25

**Authors:** Christos Eliopoulos, Dimitrios Arapoglou, Serkos A. Haroutounian

**Affiliations:** 1 Institute of Technology of Agricultural Products, Hellenic Agricultural Organization—DIMITRA (ELGO—DIMITRA), Sof. Venizelou 1, 14123 Athens, Greece; dimarap@elgo.gr; 2 Laboratory Nutritional Physiology & Feeding, Department of Animal Science, Agricultural University of Athens, Iera Odos 75, 11855 Athens, Greece; sehar@aua.gr

**Keywords:** agro-industrial byproducts, solid state fermentation, antioxidants, nutritional composition, phytochemicals, bioactive compounds

## Abstract

Agriculture and its related industries produce annually a vast amount of byproducts and waste which comprise a large proportion of global waste. Only a small percentage is managed with environmentally acceptable procedures, while a large proportion is either incinerated or discarded into nearby open fields, causing serious environmental burdens. Since these byproducts exhibit a rich nutritional and phytochemical content, they may be considered as raw materials for various industrial applications, initiating the need for the development of sustainable and eco-friendly methods for their valorization. Among the various methods considered, Solid-State Fermentation (SSF) constitutes an intriguing eco-friendly bioprocess, being suitable for water-insoluble mixtures and providing products with improved stability and depleted catabolic suppression. Thus, there are several literature studies highlighting the aspects and efficacy of SSF for improving the nutritional and phytochemical contents of diverse agro-industrial waste. The review herein aspires to summarize these literature results with a special focus on the enhancement of their antioxidant potency. For this purpose, specific keywords were used for searching multiple scientific databases with an emphasis on the most recent studies and higher impact journals. The presented data establish the usefulness and efficacy of the SSF bioprocess to obtain fermentation products with enhanced antioxidant profiles.

## 1. Introduction

The global population is expected to continuously expand and reach 10 billion by the year 2050, increasing the possibility of humanity facing problems related to food security. To meet the projected increased nutritional demand, the global primary sector and the respective food industry have to increase their production volume. This endeavor is expected to increase the volume of agro-industrial byproducts and waste [1], causing serious environmental problems. According to a recent report from the UN Agricultural and Food Organization (FAO), one third of the annually generated agricultural products and foods, accounting for 1.3 billion tons, will become waste, causing an economic loss estimated at approximately 990 billion USD [1,2,3]. It is noticeable that every year in the USA, 88 million tons of food worth 165.6 billion USD are discarded as waste [3]. In addition, the agro-food industry produces a broad variety of diverse byproducts in volumes exceeding 190 million tons per year, of which 40–50% are fruits, vegetables, roots, and tubers [4]. These byproducts are generated during all stages of their production process (harvest, storage, distribution, consumption, etc.) [4,5,6]. Accordingly, European countries produce 96 million tons of fruits and vegetables annually, an amount corresponding to 8.5% of the global production, of which 30% will not be eaten and will be disposed of as byproducts [7]. Consequently, the development and application of eco-friendly strategies for the management of these byproducts constitutes a significant challenge, since most of the currently applied methods raise serious environmental concerns.

Today, the most common practice for managing agro-industrial byproducts is to discard them into nearby open fields or incinerate them to prevent microorganism and parasite accumulation [8,9]. It is evident that both practices are anachronistic and problematic since they cause serious environmental burdens (e.g., through the emission of greenhouse gases). They also have negative social impacts since they initiate various respiratory health diseases by releasing various toxic pollutants that deteriorate air quality [10,11]. The European Commission Action Plan for Circular Economy encourages the reduction in waste and the promotion of various innovative management procedures aiming to build a sustainable and eco-friendly future [12]. Additionally, the European Parliament directive 2008/98/EC has legislated that as agro-industrial byproducts are characterized, those possessing the potential for utilization in other applications without further processing. Therefore, all endeavors concerning the adoption of these regulations must comply with the respective environmental regulations to impede adverse effects on the environment and human health.

The vast amount of agro-industrial byproducts are known to possess a rich phytochemical content and high nutritional profile, because they contain large amounts of proteins, lipids, minerals, sugars, polyphenols, and other bioactive molecules [13,14,15]. Accordingly, they may be considered as raw materials for industries such as food, pharmaceuticals, cosmetics, etc. They also constitute substrates easily assimilable by various microorganisms [12] for re-valorization by the industrial sector, in the terms of circular economy contributing simultaneously to environmental pollution reduction. With respect to their economic perspectives, their valorization in the terms of a circular economy promotes various alternative eco-friendly green practices, demonstrating the potential to reap the benefits of the generated products through the development and isolation of diverse mixtures of antioxidant compounds [3,8,16].

The Solid-State Fermentation (SSF) bioprocess constitutes an intriguing strategy capable of addressing many of the emerging environmental issues. SSF is a biotechnological process initiated by microorganisms that utilizes as substrates a broad variety of agro-industrial solid wastes. Specifically, the SSF process has the potential to transform the agro-industrial byproducts of low commercial/economic value into high added value materials such as bioactive compounds, bioplastics, and biofuels [17]. Overall, the SSF process is an efficient eco-friendly bioprocess suitable for water-insoluble mixtures and capable of providing products with improved stability and depleted catabolic suppression [18,19].

On the other hand, SSF application is associated with several potential risks, with the most important being the development of plausible contamination during the implementation of its various steps. The main cause of contamination is non-compliance with aseptic procedures, either in the form of incomplete sterilization or through the utilization of cultures contaminated with exogenous organisms. Another potential source of contamination is related to the utilization of a contaminated medium initiated either as a consequence of sterilization system failure or by the improper application of sterilization processes (e.g., time and temperature). Finally, the utilization of improper air sterilization conditions also poses a contamination risk, resulting in air system failure. It is evident that as a multifactorial procedure the SSF process is very susceptible to microbial contamination, highlighting the adoption of proper operating conditions to avoid the potential contamination risks. Consequently, the recommended conditions include the following: (a) monitoring the substrate’s water activity, (b) the utilization of increased inoculation content, (c) controlling the pH values. In addition, since during the summer period the SSF process is more susceptible to microbial contamination, due to the presence of high temperatures, the fermentation system must be properly cooled to avoid the possibility of contamination. On the other hand, the addition of salt constitutes an intriguing strategy for the prevention of microbial contamination, especially when it is applied at a range between 15 and 18%. It must be noted, however, that although the utilization of increased amounts of salt protects the fermentation system from possible contamination, it simultaneously reduces the enzyme activity [20].

The review herein aspires to summarize and present the recent literature on various aspects of the Solid-State Fermentation bioprocess with a main focus on the improvement of agro-industrial byproduct antioxidant potency.

## 2. Method

For the implementation of this comprehensive review, we have assessed a broad series of scientific databases, including Scopus, Web of Science, PubMed, and Google Scholar. The keywords used were as follows: Solid-State Fermentation, antioxidant activity/capacity, bioactive molecules, agro-industrial byproducts valorization. The respective results revealed 150 peer-reviewed papers published during the period 2000 to 2025. Herein we have included the most recent publications containing significant experimental data and published in journals with higher impact factors. No other exclusion criteria were used.

## 3. Solid-State Fermentation Process

**Figure 1:**
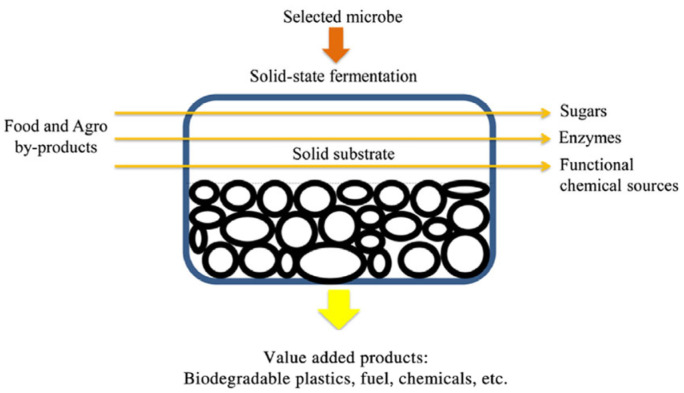
Solid-State Fermentation process application for the production of a high added value product. Modified by Manan and Webb [32].

The SSF process (Figure 1) is used for bioprocessing various agro-industrial byproducts with the aim of increasing their content of valuable functional bioactive molecules. Its efficacy is closely associated with the nature of the microorganism utilized as the initiator, the fermented byproduct, and the applied specific conditions. Fungi, yeasts, and bacteria are the most common types of initiators employed in the SSF bioprocess, with fungi and yeasts being the most frequently used. In particular, *Candida*, *Saccharomyces,* and *Aureobasidium* are the most commonly used yeast strains in the SSF bioprocess, whereas *Aspergillus*, *Penicillium,* and *Rhizopus* comprise the most suitable fungal strains, which have been used for the production of various valuable bio-products [21,22]. It must be noted, however, that recently the use of bacteria has gained interest, since they have been determined to display the capability of providing efficient bio-products when they are applied to SSF, with *Bacillus* and *Streptomyces* being the most frequently used strains [21,23,24].

The utilization of solid materials for the SSF process facilitates the action of microorganisms since it resembles their natural environment. The major advantage of the SSF process, compared with liquid fermentation, is connected to the availability of oxygen and the limited presence of unwanted organic wastewater which increases the concentration [20]. Thus, the extracts derived from the SSF bioprocess are highlighted for their high quality and activity. Another advantage of SSF is related to the absence of organic solvents, which lowers the operational costs [9,19] and the production of high added value products from low-cost agro-industrial byproducts. Consequently, the SSF process constitutes a preferred strategy compared with submerged fermentation.

The substrate is another crucial factor associated with SSF’s efficacy and is closely related to the economic value of fermentation products. Substrate selection is a highly important step, since it provides the microorganisms with the required nutrients, ensuring fermentation success. For this purpose, due to their rich nutritional content and availability, the organic byproducts derived from agricultural and agro-industrial processes along with food waste constitute ideal options for acting as SSF substrates, simultaneously reducing their environmental impact [21]. In addition, SSF efficacy is affected by various operational parameters such as substrate moisture content, particle size, pH, temperature, microorganism concentration, sterilization, and aeration [25]. With respect to the particle size, substrates consisting of small particles provide a greater fermentation surface, facilitating microbial colonization. It must be noted however, that although larger particles promote aeration, they also constrain the available microbial colonization surface [25,26,27]. On the contrary, the utilization of extremely small particles is not suitable for the SSF process, since they may result in the substrate’s agglomeration, preventing the proper transfer of oxygen. Thus, they have a negative impact on microorganism performance.

Moisture constitutes another crucial factor for the achievement of a successful SSF procedure. It must be noted that bacteria and fungi display different requirements, since the optimum moisture content for fungi fluctuates between approximately 40 and 60%, while the bacteria demand a higher moisture content ranging from 60 to 85% [24]. Nevertheless, the optimum moisture levels for the SSF bioprocess depends on the diffusion rate between the nutrients and oxygen/dioxide [28]. Thus, the high moisture presumably leads to reduced porosity that directly affects the diffusion and circulation of oxygen, whereas low moisture prevents the dissolution of nutrients, limiting the microbial growth.

Temperature is considered a potent indicator of microbial activity. During aerobic fermentation, all available oxygen is consumed by microorganisms for the decomposition of the organic molecules. Thus, an amount of free energy and heat is produced, increasing the fermentation temperature. Although high temperatures may adversely affect the microbial growth and the formation of the desired products, in some cases it also promotes the production of certain enzymes [21,29,30,31].

In summary, the SSF bioprocess is a multifactorial process characterized as a promising biotechnological procedure when applied to various agro-industrial byproducts, providing products with improved physicochemical characteristics such as an enhanced antioxidant profile.

## 4. Impact of Solid-State Fermentation on Substrate’s Antioxidant Potency

Agro-industrial byproducts, especially those produced by the food industry, are characterized as suitable raw materials for SSF bioprocess implementation because of their rich physicochemical profile composed of many nutrients and bioactive compounds. Thus, they are utilized for the production of various bioactive compounds such as phenolics, organic acids, and flavor and aroma compounds, which can be used as antioxidative, antimicrobial, anti-inflammatory and anti-allergic agents [33,34]. These products are utilized as additives into various foodstuffs with the aim of improving their stability, organoleptic and technological properties, and nutritional content for the prevention of various diseases [35].

Phenolics comprise the most significant group of bioactive compounds. These plant origin natural molecules are well adopted in the human diet due to their abundance and health-promoting properties. Their structure consists of an aromatic ring substituted by one or more hydroxy groups and they are categorized into three principal groups consisting of phenolic acids, flavonoids, and tannins [33]. Phenolics are known to possess a plethora of physiological functions such as anti-allergenic, anti-atherogenic, anti-inflammatory, antimicrobial, antioxidant, anti-thrombotic, cardioprotective, and vasodilatory activities [36].

As it was already pointed out, phenolics are abundant in most agro-industrial byproducts. A characteristic example is apple pomace, the remains of apple processing, which contains phenolics accounting for 82–99% of its phytochemicals [37]. Its utilization as a SSF substrate with *P. chrysosporium* as the initiator was investigated by Ajila et al. [38] with the aim of determining the optimum fermentation parameters for the production of phenolic antioxidants. The respective results indicated the beneficial effect of the SSF process on both the nutraceutical value and antioxidant activity of apple pomace, since the IC_50_ of the polyphenol extract of fermented apple pomace was 12.24 μg compared with the unfermented sample which was 20.12 μg. Another study [39] utilized grape pomace as the substrate and *Aspergillus* and *Penicillium* strains as the SSF initiators to increase the amount of gallic acid, a phenolic antioxidant. Similar reports for SSF application in a substrate consisting of pomegranate peel and creosote bush leaves and *A. niger* GH1 as the SSF initiator increased their ellagic and gallic acid contents considerably [40]. Recently, the cultivation of chokeberry has also gained considerable interest since it possesses a high content of phenolic compounds and considerable antioxidant potency. Consequently, chokeberry pomace was used as a substrate in SSF initiated with *A. niger* and *R. oligosporus*. The respective results are indicative of SSF’s beneficial impact on antioxidant potency and total phenolic and flavonoid content. Moreover, the production of lipids was also enhanced by improving their nutritional quality characteristics [41]. The same substrate was used in SSF with *L. edodes* as the initiator and resulted in a much higher ellagic acid content [42]. In another related experiment, Sharma et al. [43] studied the efficacy of the SSF bioprocess initiated by *A. niger* ARNU-4 for the production of gluconic acid derived from sugarcane molasses, using tea waste as the substrate. Finally, the *M. purpureus* fungus was used as a potent SSF initiator on a substrate consisting of peanut meal along with coconut residue and soybean meal for red pigment production [44].

The FAO reported that the pineapple canning industry and/or fruit consumption produce large amounts of byproducts and waste, estimated as ten tons per hectare. In this respect, Rashad et al. [45] implemented a comparative assessment of the outcome of the SSF procedure utilizing pineapple byproducts vs. their initial form by determining the antioxidant capacity and in vitro anticancer activity against various human cancer cell lines. Specifically, they used a mixture of pineapple juicing byproducts composed of the pulp, peels, skin, core, and crown as the substrate for a Solid-State Fermentation procedure initiated by *K. marxianus* NRRL Y-8281. The phytochemical and nutritional content of the fermented material was improved compared with the unfermented material, since the total phenolic content was increased to 120 mg GAE/100 g of dry weight (dw). Furthermore, the linoleic acid radical scavenging capacity was elevated to 95%, compared with the corresponding unfermented material (88%). With respect to anticancer potency, the fermented extracts were determined to be more efficient against MCF-7, A549, and HCT116 cell line activities, being comparable to that of doxorubicin.

Another literature report concerned the evaluation of the phenolic content of the SSF bioprocess using pineapple juicing byproducts enriched with soy flour extracts as the substrate. For this purpose, the antioxidant potency of the following two mixtures was exploited: one composed of pineapple byproducts and soy flour in equal proportions and a second consisting of 90% pineapple. The respective results revealed that the fermented mixture of equal amounts displayed enhanced antioxidant activity, whereas its total phenolic content was increased by 39.3% compared with the pineapple-rich mixture, which was increased by 79.4%. A similar pattern was recorded for their DPPH free radical scavenging capacity and *β*-carotene content, which displayed a significant increase in both experiments [46]. Bind et al. [47] performed a similar study using a mixture of pomegranate peels and soy flour as the SSF substrate and *A. niger* as the initiator. The respective results revealed an increase in both total phenolic content and DPPH free radical scavenging capacity, ranging from 13.21 to 15.66 μg/mL and 16.88% to 43.01%, respectively. In addition, with the aim of process optimization, they investigated the influence of media, incubation time, and pH value, eventually achieving increases in both parameters which reached 20.82 μg/mL and 46.21%, respectively. Another study investigated the SSF bioprocess using grape pomace as the substrate and *Zygomycetes* fungi, *A. elegans,* and *U. isabelline* as initiators to increase their antioxidant activity [48]. The respective results indicated that on day 4 the total phenolic content of pomace fermented with *A. elegans* increased by 47% compared with the original value of 4.78 mg GAE/g dw. This value was slightly reduced by the end of fermentation, while the utilization of *U. isabelline* resulted in a significant reduction by 27% of the phenolic content from the initial value of 0.96 mg QE/g dw. The observed increase in *A. elegans* microorganism utilization can be rationalized considering its ability to act as a lignocellulosic agent by secreting cellulolytic enzymes that exhibit the potential to break *β*-glycosidic bonds, resulting in the production of free phenolics. Unlike *A. elegans*, the reduction observed for *U. isabelline* can be justified presumably because of phenolic compound degradation and/or enzymatic polymerization [48]. Another study concerned the SSF bioprocess initiated by *R. miehei* NRRL 5282 using various substrates consisting of black grape, apple, and yellow pitahaya byproducts and targeting the exploitation of the antioxidant potency of products using freeze- and oven-drying techniques. The respective results of their total phenolic content and DPPH free radical scavenging capacity assessments indicated that the freeze-dried samples possessed higher antioxidant properties [49].

Because of their rich nutritional profiles, vinification and grape juicing byproducts comprise ideal substrates for the application of the SSF bioprocess. Thus, they have been examined as substrates in several SSF experiments using various strains such as *A. niger* GH1, PSH, Aa-20, and ESH as the initiators [39]. The antioxidant potency of all fermented samples was increased compared with the non-fermented material. The highest increase was determined for *A. niger* GH1, which recorded the highest value for both DPPH scavenging activity and total phenolic content. The same strain was used for the SSF of mango seeds [50,51] leading to a significant increase in both total phenolic content and DPPH scavenging activity for the fermented material, with the value of total phenolic content displaying a more than three-fold increase. Another study on the utilization of fig jam processing byproducts as SSF substrates concerned the comparable utilization of the following four different microorganisms, *R. oryzae* (PP4-UAMI), *Trichoderma* sp., *A. niger* HT4, and *A. niger* GH1, as the initiators [52]. The results indicated that all fermented products displayed similar antioxidant profiles. In addition, this study also revealed the importance of fermentation parameters such as mineral composition, pH, temperature, and moisture in SSF efficacy since the highest antioxidant potency was observed for 36 h and 60 h of fermentation using the *A. niger* HT4 and *A. niger* GH1 strains as the initiators, respectively. In another study, grapefruit byproducts fermented at 50% and 70% moisture, using Raimbault columns as bioreactors of the SSF bioprocess initiated by *A. niger* GH1 [53], presented a similar antioxidant pattern. Specifically, their DPPH and Ferric Reducing Antioxidant Power (FRAP) values were reduced during the first 24 h and then increased until the end of fermentation (120 h), with the sample containing 70% moisture displaying the highest antioxidant profile. Duff et al. [54] exploited a SSF procedure using plum byproducts mixed with brandy waste as the substrate and *A. niger* and *R. oligosporus* as the initiators. The respective results revealed an increment of total flavonoids and phenolics for both microorganisms, with *R. oligosporus* recording the highest values. A similar pattern was observed for the total phenolic content, with *A. niger* and *R. oligosporus* recording 21% and 30% increments, respectively. In addition, SSF exhibited a positive impact on their antioxidant capacity, since the DPPH scavenging activity was increased by 27.70% for the sample fermented with *A. niger* and 35.40% for the fermentation with *R. oligosporus*. A similar study was conducted for apricot pomace using the same initiators. The results indicated that the total phenolic content was increased by 78% and 34% for *R. oligosporus* and *A. niger*, respectively. A similar behavior was observed for their total flavonoid content with a 38% and 12% increase for *R. oligosporus* and *A. niger*, respectively. Finally, the DPPH assay which is considered an indicator of antioxidant potency displayed an 18% increment for both strains [55].

With respect to the incorporation of a nitrogen source into SSF substrates, Vattem and Shetty [56] added ammonium nitrate (NH_4_NO_3_) or Fish Protein Hydrolysate (FPH) to a cranberry pomace substrate and used *R. oligosporus* as the initiator. The respective results revealed enhanced *β*-glucosidase activity for both additives, whereas the DPPH assay displayed a 5% increment only for the ammonium nitrate. Finally, both total phenolic content and the antioxidant activity were elevated for both treatments. In another experiment, utilizing the *R. stolonifer* LAU 07 strain for SSF with substrates composed of cocoa pod husk, cassava peel, and palm kernel cake, all byproducts of Nigeria’s agricultural sector, showed a higher antioxidant capacity for all fermented substrates with respect to their DPPH free radical scavenging capacity and IC_50_ (mg/mL) values. Additionally, the nutritional value, evaluated as crude protein, crude fiber, ash, and lipid content, was considerably improved in all cases [57]. Another study concerning the utilization of cocoa shells that originated from cocoa processing as the SSF substrate resulted in a considerable increase in the total phenolic content, reducing activity and free radical scavenging activity. The authors reported that all examined parameters were increased at the end of the fermentation period presumably because the fermented products were capable of depleting DPPH free radical scavenging capacity by 50–70%. Conversely, their total anthocyanin and flavonol levels remained intact [58].

The utilization of orange pomace as the substrate for studying the SSF bioprocess using *P. variotii* strain as the initiator, with the aim of producing tannase and phytase enzymes, was studied by Maderia et al. [59]. The antioxidant capacity evaluation of the fermentation outcome revealed the efficacy of SSF, since the antioxidant profile of orange pomace displayed a 10-fold increase. Kaur et al. [60] utilized a mixture of fruit and vegetable byproducts as the SSF substrate, aiming to enhance their *β*-carotene content. For this purpose, they studied the SSF bioprocess initiated by *B. trispora* (+) MTCC 884 using a mixture of orange, carrot, and papaya peels as the substrate. The final goal of the study was to investigate the influence of various parameters such as pH, temperature, nitrogen sources, and incubation time. The obtained results showed that fermentation lasting 96 h, at 30 °C, and 6.2 pH provided the best results with respect to *β*-carotene production, since a sharp increase in its content was observed along with an increase in the antioxidant potency. The authors also reported that the elevated amount of *β*-carotene is responsible for the detected improved scavenging activity, confirmed through DPPH assays, for a period exceeding 90 days. Nemes et al. [61] revealed the positive impact of oat bran acid pretreatment on the SSF bioprocess. Specifically, its incorporation in SSF with *A. niger* as the initiator showed that on the sixth day of fermentation the total phenolic content and the vanillic acid amount displayed a notable increment, while the highest activity for the DPPH assay was recorded on day four of the process (83.33%).

Oregano byproducts are ideal candidates for the SSF bioprocess due to their rich nutritional profile and bioactive compound content. Their incorporation in the SSF bioprocess initiated by the lactic acid bacterium (LAB) *L. mesenteroides* increased their total phenolic and flavonoid contents, along with the antioxidant activity which was increased compared with the initial samples [62]. Zhang et al. [63] investigated the incorporation of highland barley bran in a SSF bioprocess initiated by *B. subtilis.* The authors determined the following SSF conditions as optimal for polyphenolic content enhancement: 10% inoculum, liquid–feed ratio of 1.80, fermentation temperature of 30 °C, and a 93.5 h fermentation time. It must be noted that all fermented samples displayed a higher polyphenolic content compared with the unfermented material, while a similar observation was made for their DPPH free radical scavenging activity, Fe ion reducing capacity (FRAP), and hydroxyl radical scavenging, which also increased. In another experiment, Ordonez-Cano et al. [64] performed the SSF bioprocess with a pistachio green hull substrate and using *A. niger* GH1 as the initiator, aiming to enhance the amount of contained phenols. For this purpose, they determined the optimum fermentation parameters, verifying that at the end of fermentation the antioxidant profile was improved. The determined values for the assessment of total phenolics, 2’-azino-bis(3-ethylbenzothiazoline-6-sulfonic acid) (ABTS), DPPH, and FRAP were increased considerably by 129, 71, 124, and 1039%, respectively. Another SSF study carried out on cocoa pod husk with *R. stolonifera* as the initiator targeted the improvement of the nutritional profile and antioxidant potency. The efficacy of the method was confirmed by performing Oxygen Radical Absorbance Capacity (ORAC) and DPPH assays, which verified a considerable increase in antioxidant activity [65]. Finally, Du et al. [66] examined the utilization of *Glycyrrhiza* stems and leaves as substrates for the SSF bioprocess initiated by a mixture of *B. subtilis*, *L. plantarum,* and *S. cerevisiae* at a ratio of 1:1:1. The obtained results revealed the positive impact of SSF, since the fermentation outcome recorded an increased flavonoid content and enhanced scavenging activities for the DPPH radical, hydroxy radical, and reducing power assays.

It is evident that the SSF process constitutes an eco-friendly and sustainable approach, which may be applied to the management of a plethora of agro-industrial byproducts and wastes, providing products with enhanced antioxidant potency. According to the presented herein literature results, the application of the SSF bioprocess is capable of increasing the total phenolic content and the antioxidant profile of diverse byproducts. SSF’s efficacy depends on various parameters, such as the microorganism used as the initiator, the material used as the substrate, and the applied specific conditions. Several endeavors have been implemented concerning the investigation of SSF performance and for the determination of its optimum conditions. All relevant results indicated that it is feasible through optimization to obtain a larger amount of antioxidant molecules and an enhanced antioxidant profile. Table 1 summarizes the effects of the SSF process concerning all abovementioned studies.

## 5. Future Challenges for the Solid-State Fermentation Bioprocess

The SSF procedure is considered a sustainable, green, and eco-friendly viable tool which could be widely applied to address an emerging problem concerning the environmentally sound management of agro-industrial wastes. In addition, SSF is applied to the production of various mixtures of bioactive compounds [67,68]. Despite its advantages, the SSF procedure must overcome a plethora of challenges in order to scale up and be widely applicable on a large scale. The main challenges that should be addressed concern biomass estimation, heat transfer, recovery, operational control, and reproducibility and heterogeneity in the case of diverse natural matrices utilization [69,70,71].

A significant obstacle associated with the industrial-scale application of SSF is connected to the proper management of various significant limitations associated with heat and mass transfer in solid organic matrices, since it is established that they affect several crucial factors of the procedure. Among them, temperature is the most crucial parameter since a prerequisite for successful SSF implementation is the utilization of various microorganisms that operate under specific temperature environments. During SSF bioprocess implementation, the temperature records an increment which should be regulated, especially for organic materials which are characterized by their low thermal resistance [72,73]. For this purpose, temperature is usually adjusted by applying a forced aeration system that is capable of removing the excess heat and preventing unwanted thermal fluctuations. On the other hand, this technique may have adverse effects through the reduction in moisture content and the creation of a drier environment [74]. For this purpose, it is necessary to develop suitable bioreactors equipped with both aeration and cooling systems.

The bioreactors which are most frequently used today for SSF performance are categorized as the following types: (a) tray bioreactors, (b) packed-bed bioreactors, (c) rotating drum (or stirred-drum) bioreactors, (d) fluidized-bed bioreactors, (e) instrumented lab-scale bioreactors. Tray bioreactors are the most frequently used bioreactors consisting of a chamber with a stable temperature and passive aeration [75]. The packed-bed bioreactors are usually preferred for fungal cultures mainly because they are capable of facilitating a substrate’s growth and decomposition, producing thus higher yields. Their main disadvantage concerns heat accumulation which results in a considerable increase in the temperature during the process, reducing the growth of the microorganism and leading to a low production yield [76,77]. The bioreactors with rotating (or stirred) drums are capable of providing smooth and uniform mixing, thus promoting the design of baffles and heat reduction. These bioreactors are not equipped with an agitator within the substrate bed and are usually preferred for biofuel production using cellulosic materials as the substrates. They are connected to the creation of agglomeration, along with the difficulty in controlling the heat and mass transfer inside the equipment [77]. The fluidized-bed and instrumented lab-scale bioreactors comprise two additional types of bioreactors used in SSF. The fluidized-bed bioreactor allows the independent movement of particles [78], whereas the lab-scale bioreactor is mainly used for monitoring the operational condition effects using an online automated monitoring and control system [79,80].

Another important step for the industrial application of SSF concerns the selection of an appropriate substrate consisting of a mixture of byproducts, since it is also implicated with the resolution of the issues connected to mass (and heat) transfer. It is evident that a properly selected homogeneous substrate is capable of maintaining stable thermal parameters and facilitating the transport of the gas or liquid interface. On the other hand, a heterogeneous medium makes it difficult for microorganisms to access substrates consisting of a different nutritional profile [31]. Thus, the byproducts’ mixture should be selected after careful design, to avoid adverse effects on porosity which leads to the poor colonization of microorganisms in the substrate [81].

Despite the abovementioned limitations, the SSF process constitutes a bioprocess displaying the potential of producing a broad variety of diverse commercial and industrial products such as enzymes, secondary metabolites, biofuels, aroma compounds, organic acids, biopolymers, biosurfactants, pigments, etc. Specifically, the SSF procedure is being applied at industrial scale for the (a) production of biofuels from biomass in the context of biological delignification through the biological deconstruction of plant biomass, (b) bioremediation of dye residues derived from textile industries, (c) detoxification of agro-industrial byproducts such as Jatropha cake. In this context, Thomas et al. [82] have published a comprehensive review concerning the current developments for the utilization of solid-state fermentation to produce various industrial products.

Overall, despite the emerging challenges closely connected to its industrial-scale performance, the SSF bioprocess can be characterized as a viable and environmentally sustainable approach to produce high added value bioactive compounds. Thus, there is an urgent need for further research towards the definition of crucial parameters affecting the successful implementation of the SSF process with respect to the applied aeration, cooling, and mixing technologies combined with the proper regulation of the thermal and mass parameters. Their definition, along with the proper design of novel bioreactors, will facilitate the optimization and efficacy of this bioprocess and will pave the way for its scaling-up and commercialization.

## 6. Conclusions

Although agro-industrial byproducts are characterized as raw materials because of their physicochemical profiles, their currently applied management methods have negative environmental impacts, consisting of their disposal into nearby open fields or insanitation. Thus, there is an emerging need for the development and application of environmentally sound sustainable green technologies for their management. On the other hand, the nutritional content and antioxidant compounds of these byproducts distinguishes them as a significant source of various plant origin antioxidants and therefore an intriguing valorization subject. In this regard, the environmentally friendly Solid-State Fermentation (SSF) bioprocess is considered a promising candidate, since it is capable of transforming them into high added and nutritional value products, ensuring their economically viable management through valorization.

The main elements affecting the efficacy of the SSF procedure, including microbial strains, substrate composition, process duration, temperature, and moisture, were investigated and optimized in the context of numerous research endeavors targeting the production of a broad variety of high added value bioactive natural compounds.

The main objective of this review was to summarize and present the most recent literature results concerning the application of the SSF bioprocess on various agro-industrial wastes with the aim of obtaining enhanced antioxidant capacities and presenting their potency for industrial valorization. The results presented herein establish the usefulness and efficacy of SSF to function as a valuable and eco-friendly bioprocess that enhances the antioxidant potency of various substrates through the determination of increased values for the total phenolic content of all fermented byproducts and the respective outcomes of DPPH, ABTS, FRAP, and ORAC assays.

## Figures and Tables

**Table 1:** Effect of Solid-State Fermentation on antioxidant profile.

By-Product	Microorganism	Antioxidant Parameters	References
Apple pomace	*P. chrysosporium*	Increased antioxidant activity, reduced IC_50_	[38]
Grape byproducts	*Aspergillus* and *Penicillium* strains	Increased gallic acid yield	[39]
Pomegranate peel and creosote bush leaves	*A. niger* GH1	Enhanced production of ellagic and gallic acids	[40]
Chokeberry pomace	*A. niger* and *R. oligosporus*.	Elevated total phenolic and total flavonoid content	[41]
Chokeberry pomace	*L. edodes*	Enhanced ellagic acid content	[42]
Tea waste	*A. niger ARNU-4*	Gluconic acid production	[43]
Peanut meal, coconut residue, and soybean meal	*M. purpureus*	Red pigment production	[44]
Pineapple byproducts	*K. marxianus* NRRL Y-8281	Increased phenolic content and potential anticancer activity	[45,50]
Pineapple byproducts enriched with soy flour		Enhanced antioxidant activity and phenolic content; DPPH’s free radical scavenging capacity and *β*-carotene content recorded a significant increase	[46]
Pomegranate peels and soy flour	*A. niger*	Increased value for total phenolic content and improved DPPH free radical scavenging activity	[47]
Grape pomace	*A. elegans* and *U. isabelline*	Increased total phenolic content	[48]
Black grape, apple, and yellow pitahaya byproducts	*R. miehei* NRRL 5282	Higher antioxidant activity	[49]
Grape byproducts	*A. niger* GH1, PSH, Aa-20, and ESH	Improved DPPH scavenging activity and total phenolic content	[39]
Fig byproducts	*R. oryzae* (PP4-UAMI), *Trichoderma* sp., *A. niger* HT4, and *A. niger* GH1	Increased total phenolic content	[52]
Grapefruit byproducts	*A. niger* GH1	Increased DPPH and FRAP activity	[53]
Plum byproducts	*A. niger and R. oligosporus*.	Elevated total flavonoids and phenolic content and enhanced DPPH’s capacity	[54]
Apricot pomace	*R. oligosporus* and *A. niger*	Higher total phenolic and total flavonoid content and improved DPPH’s activity	[55]
Cranberry pomace	*R. oligosporus*	Enhanced *β*-glucosidase activity and DPPH assay total phenolic and antioxidant activity for both conditions	[56]
Cocoa pod husk, cassava peel, and palm kernel cake	*R. stolonifer* LAU 07	Increased DPPH free radical scavenging activity	[57]
Cocoa shells	*R. stolonifer*	Elevated total phenolic compounds, reducing activity and free radical scavenging activity	[58]
Orange pomace	*P. variotii*	Enhanced antioxidant profile	[59]
Orange, carrot, and papaya peels	*B. trispora* (+) MTCC 884	Increased *β*-carotene’s production	[60]
Oat bran	*A. niger*	Higher total phenolic content and vanillic acid, improved DPPH’s free radical scavenging capacity	[61]
Oregano byproducts	*L. mesenteroides*	Increased total phenolics, flavonoids, and antioxidant activity	[62]
Highland barley bran	*B. subtilis*	Elevated polyphenol concentration, DPPH radical scavenging activity, Fe ion reducing capacity, and hydroxyl radical scavenging activity	[63]
Pistachio green hull	*A. niger* GH1	Enhanced phenolics, ABTS, DPPH, and FRAP	[64]
Cocoa pod husk	*R. stolonifer*	Higher ORAC and DPPH assays	[65]
*Glycyrrhiza* stems and leaves	*B.*, *L. plantarum,* and *S. cerevisiae* mixed at a ratio of 1:1:1.	Increased scavenging activities of DPPH radical, hydroxyl radical, and reducing power	[66]

## Data Availability

The data are contained within the article.
